# Enhancing amantadine delivery through PLGA micelles: a novel approach using oleic acid and Pluronic F68 for sustained release and reduced toxicity

**DOI:** 10.1039/d5ra05261k

**Published:** 2025-11-04

**Authors:** Ismail A. Adwibi, Swaroop Chakraborty, Bashiru Ibrahim, Hanene Ali-Boucetta, Eugenia Valsami-Jones

**Affiliations:** a School of Geography, Earth and Environmental Sciences, College of Life and Environmental Sciences, University of Birmingham Birmingham B15 2TT UK e.valsamijones@bham.ac.uk; b Nanomedicine, Drug Delivery & Nanotoxicology (NDDN) Laboratory, School of Pharmacy, College of Medical and Dental Sciences, University of Birmingham Birmingham B15 2TT UK

## Abstract

Micelles are emerging as effective drug delivery carriers. This study presents the encapsulation of amantadine, a treatment for Parkinson's disease, into poly (lactic-*co*-glycolic acid) (PLGA) micelles using Pluronic F68 and oleic acid (OA) surfactants. These surfactants were selected for their ability to control drug release kinetics and protect the drug from enzymatic degradation. Dynamic light scattering (DLS) revealed that the prepared Amantadine/OA-Pluronic F68-PLGA micelles had a uniform hydrodynamic diameter of 182.4 ± 3.4 nm with a low polydispersity index (PDI) of 0.137. Transmission electron microscopy (TEM) confirmed the spherical structure, with a core–shell configuration: the inner hydrophobic core composed of oleic acid, polypropylene oxide (PPO), and poly(lactic-*co*-glycolic acid (PLGA), and an outer hydrophilic shell of polyethylene oxide (PEO) and glycolic acid (GA). Fourier-transform infrared (FTIR) spectroscopy indicated a decreased intensity of the carbonyl (C=O) stretching band at 1750 cm^−1^, while the C–O–C stretching peak at 1050 cm^−1^ remained unchanged. Additional functional group peaks at 1450 cm^−1^ (–CH_3_) and 1226 cm^−1^ (C–N) further supported the micelle formation and amantadine encapsulation. *In vitro* cytotoxicity assays (such as MTT and SRB) showed a concentration-dependent reduction in J774 cell viability, with toxicity reduced to below 10% when amantadine was encapsulated. This formulation enables controlled release, potentially reducing administration frequency while extending therapeutic efficacy. The novelty of this study lies in the combination of Pluronic F68 and oleic acid surfactants, enhancing drug stability and delivery efficiency, making it a promising approach for improving amantadine delivery in Parkinson's treatment.

## Abstract 2

A dual-surfactant system of oleic acid and Pluronic F68 with PLGA creating stable micelles for the sustained release and reduced cytotoxicity of amantadine.

## Graphical abstract

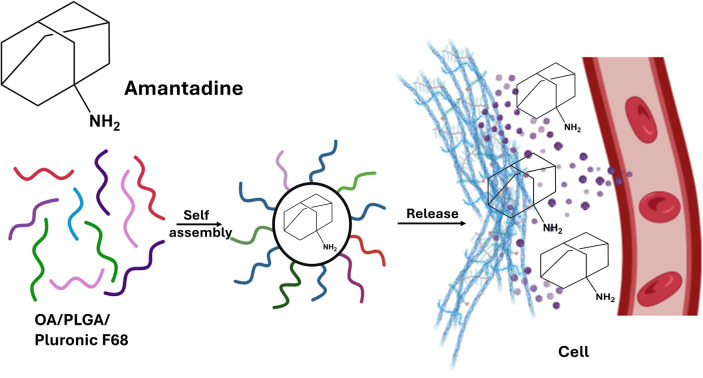

## 1. Introduction

Polymeric micelles are gaining recognition as a versatile and efficient drug delivery system, offering significant advancements in pharmaceutical sciences. These nanocarriers enhance therapeutic outcomes by improving drug solubility, protecting drugs from enzymatic degradation, and enabling targeted delivery while minimizing systemic toxicity.^1^ The unique structure of polymeric micelles, composed of amphiphilic block copolymers, makes them suitable for encapsulating both hydrophilic and hydrophobic drugs.^2^ They have been widely investigated for the treatment of diseases such as cancer,^3^ cardiovascular disorders,^4^ neurological conditions^5^ and infectious diseases.^6,7^ By encapsulating drugs within micelles, researchers can achieve sustained drug release, reduced side effects and enhanced drug stability.^8^

Parkinson's disease (PD) is a progressive neurodegenerative disorder caused by the loss of dopamine-producing neurons in the brain's substantia nigra.^9–13^ Amantadine, an antiviral drug, has shown therapeutic benefits for PD by antagonizing *N*-methyl-d-aspartate (NMDA) receptors. Although its precise mechanism of action remains unclear, amantadine has several pharmacodynamic actions to improve motor symptoms and reduce levodopa-induced dyskinesia.^14^ Previous studies indicated that amantadine boosts dopamine signalling through both pre and post synaptic pathways. Amantadine also enhances dopamine production, turnover, uptake and release from striatal dopaminergic terminals, and increases striatal l-amino acid decarboxylase activity, which supports dopamine synthesis in the brain. It also strengthens the response to dopamine (D2) receptor stimulation by apomorphine, and has been reported to raise D2 receptor binding in rodents. Moreover, amantadine may modulate monoamine oxidase B (MAO-B) activity in rodents.^15^ While extended-release amantadine formulations have been approved by the FDA, they are associated with slow absorption rates and a short half-life, limiting their effectiveness.^16^

To overcome these limitations, polymeric micelles offer an attractive approach for encapsulating amantadine, allowing for improved drug solubility, stability, and controlled release. Poly (lactic-*co*-glycolic acid) (PLGA) is one of the most widely used biodegradable polymers in micelle formulations due to its excellent biocompatibility and safety profile.^17,18^ Furthermore, the use of surfactants such as Pluronic F68 and oleic acid can prevent aggregation and improve micelle formation, stability, and drug loading capacity. The self-assembly of these surfactants into micelles enhances their ability to encapsulate drugs and facilitate sustained release.^19^

**Fig. 1:**
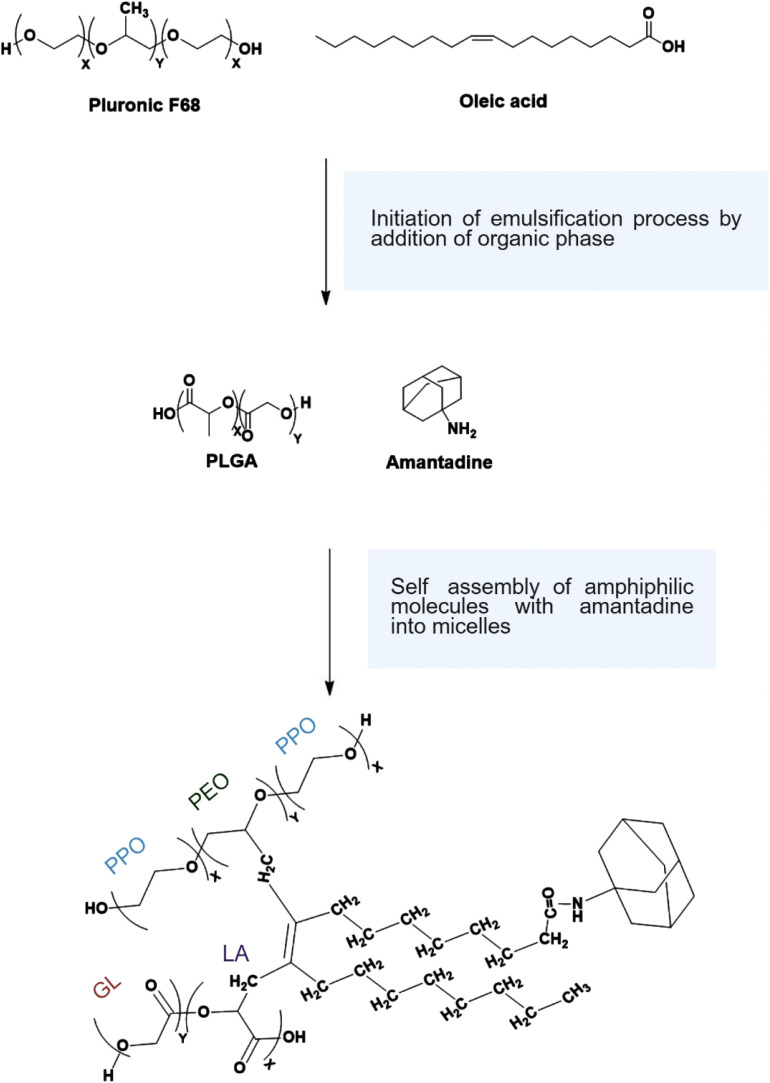
Schematic representation of the encapsulation of amantadine within self-assembled amphiphilic block copolymer micelles, facilitated by the anionic surfactants' oleic acid and Pluronic F68. The surfactants enhance micelle stability and drug loading by forming a core–shell structure. The hydrophobic core consists of oleic acid, polypropylene oxide (PPO) from Pluronic F68, and lactic acid (LA) from PLGA, surrounding the amantadine. The outer shell, formed by the hydrophilic segments of Pluronic F68 (polyethylene oxide, PEO) and glycolic acid (GL) from PLGA, provides stability in an aqueous environment, enabling sustained and controlled drug release.

The novelty of this study lies in the use of Pluronic F68 and oleic acid to encapsulate amantadine within PLGA micelles (Fig. 1). This dual-surfactant system has not been reported before and is expected to improve micelle stability, increase drug loading capacity, and offer a controlled and sustained drug release profile. These advantages could significantly enhance the therapeutic efficacy of amantadine in Parkinson's treatment by addressing its pharmacokinetic limitations. This study provides a novel formulation that could lead to more effective treatments with reduced side effects, offering a promising solution for advancing drug delivery systems in neurological disorders.

## 2. Materials and methods

### 2.1. Materials

Amantadine hydrochloride (C_10_H_17_N·HCl), poly (d, l-lactide-*co*-glycolide) (PLGA) with a lactide ratio of 50 : 50 (mol wt 30 000–60 000), acetone, Pluronic® F-68, glutaraldehyde prepared in 0.1 M phosphate-buffered saline (PBS), oleic acid (CH_3_(CH_2_)_7_CH=CH(CH_2_)_7_COOH), and 3-(4,5-dimethylthiazol-2-yl)-2,5-diphenyltetrazolium bromide (MTT) were purchased from Sigma Aldrich (Gillingham, Dorset, UK). The cell culture media, Dulbecco's Modified Eagle Medium (DMEM), fetal bovine serum (FBS), penicillin/streptomycin, PBS, sulforhodamine-B assay kit, and dimethyl sulfoxide (DMSO) were procured from Thermo Fisher Scientific (UK).

### 2.2. Preparation of self-assembled micelles loaded with amantadine

Self-assembled polymeric micelles were prepared by dissolving 75 mg of PLGA and 2.5 mg of amantadine in 5 ml of acetone to form the organic phase. This phase was then added to a mixture of 15 ml of oleic acid and Pluronic F-68. The solution was stirred gently overnight at room temperature to allow for acetone evaporation. After 24 hours, the micelles became visible. Unencapsulated amantadine and reactants was subjected to centrifugation at 7000 rpm and 20 °C for 1 hour using a centrifugal system (Centrifuge 5430/5430 R – High-Speed Centrifuge). The supernatant was analyzed by UV-Vis spectroscopy, which confirmed the absence of free amantadine and other reactants. The pellet was washed, resuspended in fresh medium and filtered through a 1.0 μm cellulose nitrate membrane to ensure the purity of the final micelle formulation. The resulting micelles were stored at room temperature for further characterization. The amantadine loading amount in micelles was measured at (2.457 mg) 98.28% w/w (SI).

### 2.3. Characterization of self-assembled micelles

The particle size, zeta potential, and polydispersity index (PDI) of the prepared amantadine/OA-Pluronic F68-PLGA micelles were measured using a Zetasizer Nano ZS (Malvern Instruments Ltd, Worcestershire, UK) (Table 1). Samples (100 μL, 1 mg L^−1^) were dispersed in ultrapure water (UPW), sonicated, filtered through 0.45 μm syringe filter, and analyzed using a refractive index of 1.47.^20^ Measurements were taken at 0, 24, 48, and 72 hours at room temperature. Transmission electron microscopy (TEM) was used to study the micelle morphology and size distribution. A single drop of the micelle solution was placed on a Formvar/carbon grid, dried overnight, and examined with a TEM (JOEL 1400, University of Birmingham) at an accelerating voltage of 80 kV. The molecular structure and atomic-level interactions of the micelles were analyzed by Fourier-transform infrared (FTIR) spectroscopy and nuclear magnetic resonance (NMR). The sample was suspended in deuterated chloroform (CDCl_3_) at a concentration of 1 mg L^−1^, and 600 μL was placed in an NMR tube (Kimble Chase, 500 MHz). NMR characterization was conducted using a Bruker 400 MHz NMR spectrometer with a 5 mm BBFO “smart” probe and an autosampler. FTIR spectra were collected using a Spectrum Two spectrometer (PerkinElmer) in attenuated total reflectance (ATR) mode across a range of 600–4000 cm^−1^ to detect changes in molecular bonds and functional groups.

**Table 1:** DLS measurements for the prepared Pluronic F68 – Amantadine/OA – PLGA micelles

Time (hour)	Particle size (nm) ± Stdev.	Zeta potential (mV)	PDI
0	183.1 ± 2.7	−35.2 ± 1.7	0.151 ± 0.01
24	172.1 ± 5.2	−28.6 ± 2.4	0.118 ± 0.01
48	189.4 ± 3.7	−31.8 ± 1.8	0.141 ± 0.02
72	185.1 ± 2.0	−33.9 ± 1.6	0.136 ± 0.01
Mean ± Stdev.	182.4 ± 3.4		0.137 ± 0.01

### 2.4. Encapsulated amantadine release study

Amantadine release rates from loaded micelles were evaluated by placing 2 ml of sample (0.5 mg ml^−1^) in 50 ml of phosphate buffer (pH = 7.4 and pH 5.0), at 37 °C with continuous stirring (140 rpm). At each time point 0, 1, 2, 3, 5, 10, and 21 days, 1 ml of each sample was taken out of the PBS buffer and replaced with similar fresh buffer.^5^ The release rates were determined by measuring the absorbance of amantadine at 200 nm, PLGA and amantadine-loaded micelles at 350 nm. This experiment was conducted in triplicate (*n* = 3).

### 2.5. *In vitro* cytotoxicity assay

The J774 cell line (macrophages) was purchased from the American Type Culture Collection (ATCC). Cells were maintained in DMEM supplemented with 10% FBS and 1% penicillin/streptomycin at 37 °C in a 5% CO_2_ humidified atmosphere. Cell viability was assessed using MTT and sulforhodamine-B (SRB) assays described by.^21–23^ Briefly, J774 macrophage cells were seeded at a density of 4.5 × 10^4^ cells per well in 96-well plates and incubated overnight at 37 °C in a 5% CO_2_ environment. The media was replaced with fresh media containing varying concentrations (0–30 μg L^−1^) of the amantadine/OA-Pluronic F68-PLGA micelles and incubated for 24 hours. For the MTT assay, 20 μL of MTT reagent was added to each well, followed by a 4-hour incubation. Formazan crystals observed by formation of purple colour solution, and the solution was removed. DMSO (100 μL) was added for 10–30 minutes to dissolve the crystals. Absorbance was measured at 595 nm using a TECAN Spark® multimode microplate reader, with untreated cells used as the control. For the SRB assay, SRB dye, which binds to cellular proteins under acidic conditions, was used to quantify viable cells. After a 24-hour incubation with various concentrations of micelles, 100 μL of fixation reagent was added to each well and incubated at 4 °C for 1 hour. Plates were washed three times with deionized water and dried. SRB dye (100 μL) was added to each well and stained for 2 hours at room temperature. The excess dye was removed, and wells were washed with 0.1 M acetic acid. Dried wells were solubilized with 150 μL of SRB buffer, and optical density was measured at 590 nm, with a reference filter at 690 nm.

### 2.6. Statistical analysis

All cytotoxicity assays were performed in triplicate (*n* = 3). Results are presented as mean ± standard deviation (Stdev). Statistical significance was determined using ANOVA, with **p < 0.01 and ***p < 0.001 compared to the control (untreated cells). Cell viability was calculated using the following formula:% cell viability = Absorbance of treated cells/Absorbance of controlled cells × 100

NMR spectral data were analyzed using MestreNova x64 software, while micelle sizes were measured using ImageJ software (Version 1.54j).

## 3. Results and discussion

The incorporation of amphiphilic molecules, OA and Pluronic F68, into PLGA contributes to the formation of a stable micellar structure and enables efficient loading of amantadine within the micellar core through hydrophobic interactions. DLS and TEM confirmed a uniform size distribution. The successful encapsulation of amantadine was indicated by analyzing functional group characteristics and molecular interactions using FTIR and ^1^H NMR spectroscopic techniques.

### 3.1. Structural characterisation of Amantadine/OA – Pluronic F68 – PLGA micelles

**Fig. 2:**
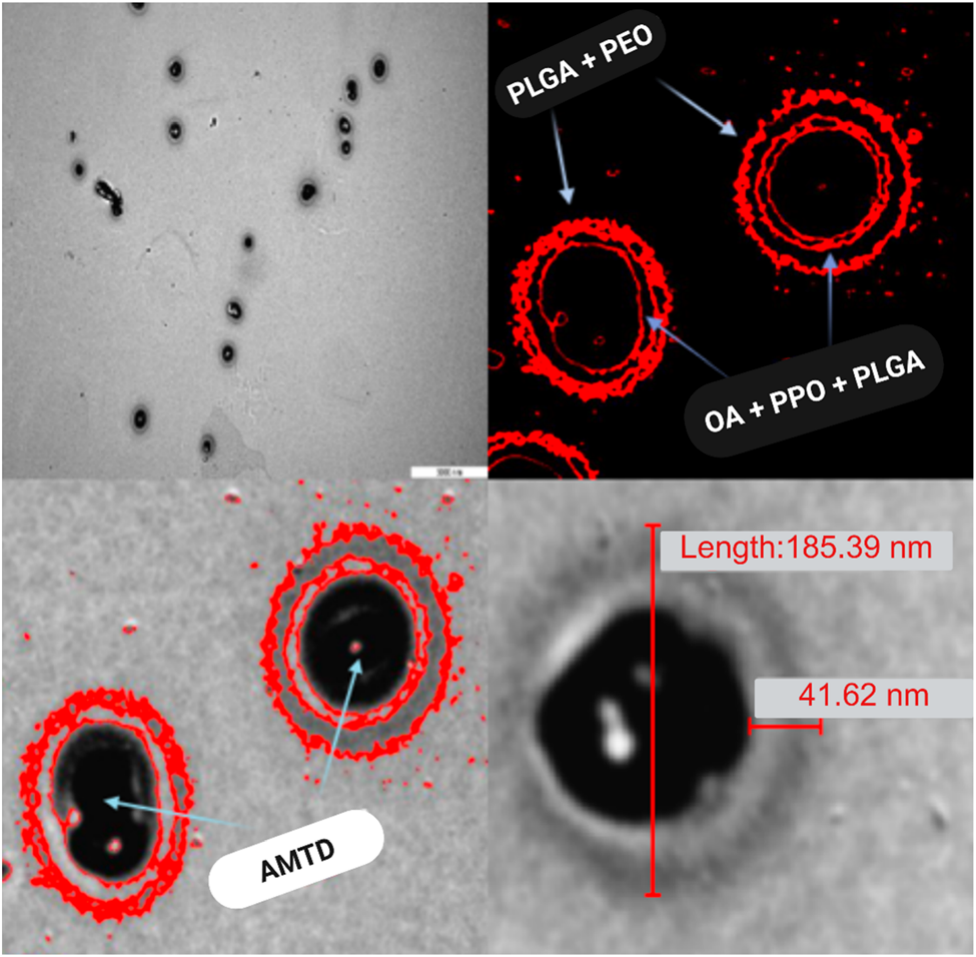
TEM images of amantadine (AMTD) encapsulated into oleic acid (OA) – Pluronic F68 – PLGA micelles. False-coloured (using ImageJ software) red circles surrounding amantadine indicate the external and internal shells of the micelle. Top left: general view of the micelles at 5000 nm scale. Top right: image depicting micelle structure, where poly (lactic-*co*-glycolic acid) (PLGA) and polyethylene oxide (PEO) represent the outer shell (the hydrophilic part of the micelles) and oleic acid (OA), polypropylene oxide (PPO), and poly (lactic-*co*-glycolic acid) (PLGA) represent the inner shell (the hydrophobic part of the micelles). Bottom left: image highlighting the amantadine (AMTD) core encapsulated into the micelles. Bottom right: image illustrating the shell thickness and size of formulated micelles 41.62 nm and 185.39 nm, respectively.

Transmission electron microscopy (TEM) provided insight into the structural organization of the amantadine-loaded micelles (Fig. 2). The micelles exhibited a smooth, spherical morphology with a well-defined core–shell structure, measuring an average diameter of 185.39 nm, as confirmed by ImageJ software. The external shell, approximately 41.62 nm thick, represents the encapsulating matrix formed by hydrophobic interactions among oleic acid (OA), Pluronic F68 (PPO) and PLGA. This core–shell configuration is essential for maintaining the stability of the micelles in aqueous environments, facilitating the encapsulation of amantadine within the hydrophobic core. The spherical and uniform dispersion of micelles is critical for ensuring predictable drug release kinetics, which is essential for controlled drug delivery applications.^24,25^ Dynamic light scattering (DLS) confirmed the size and polydispersity index (PDI) of the micelles at various time intervals (0, 24, 48, and 72 hours) (Table 1). The average hydrodynamic diameter of the amantadine-loaded micelles was measured to be 182.4 ± 3.4 nm, and the PDI remained low at 0.137, indicating a uniform size distribution. The relatively consistent size measurements over 72 hours suggest that the micelles were stable during this period, a crucial feature for drug delivery applications, as it ensures sustained and controlled release without premature degradation or aggregation.

**Fig. 3:**
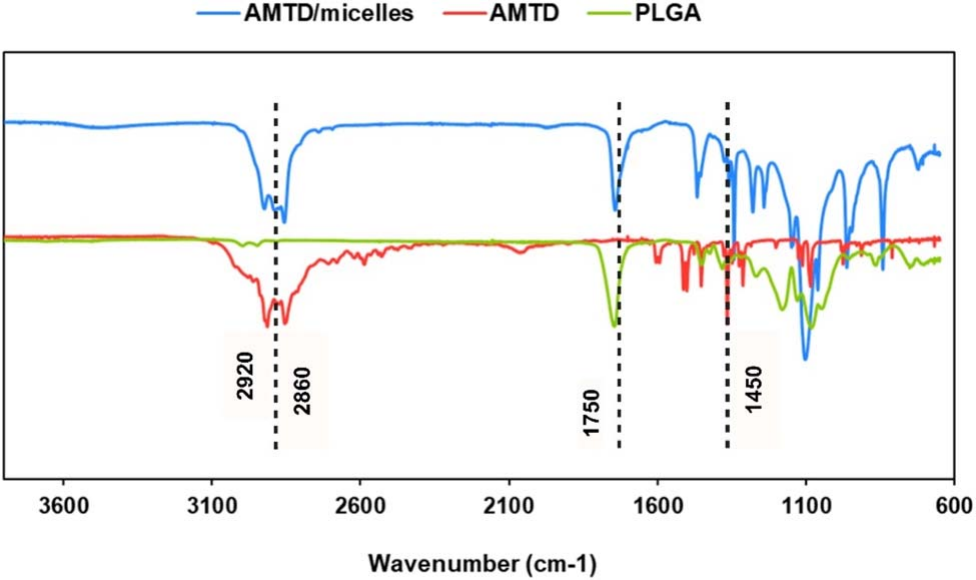
FTIR spectra collected from dried powders and showing, from bottom to top, the main functional groups of amantadine, PLGA and the encapsulated amantadine into PLGA micelles.

Fourier-transform infrared (FTIR) spectroscopy provided further confirmation of successful encapsulation by identifying characteristic functional groups. Fig. 3 presents evidence for the formation of micelles based on functional group characteristics and variations in molecular vibration features, such as stretching and bending, of the amantadine and PLGA molecules. Notably, the intensity of the carbonyl (C=O) stretching peak at 1750 cm^−1^ decreased after encapsulation, indicating an interaction between the drug and the micelle-forming materials (Fig. 3). The unchanged C–O–C stretching peak at 1050 cm^−1^ in both the micelles and PLGA suggests that the polymeric backbone remained intact during the formulation process. The peaks observed at 2860 cm^−1^ and 2920 cm^−1^ correspond to the (–CH) stretching vibrations of PLGA, oleic acid, and Pluronic F68, further confirming the presence of these materials in the micelle structure. The bands at 1450 cm^−1^ (–CH_3_) and 1226 cm^−1^ (C–N) are indicative of successful encapsulation of amantadine within the micelles.

**Fig. 4:**
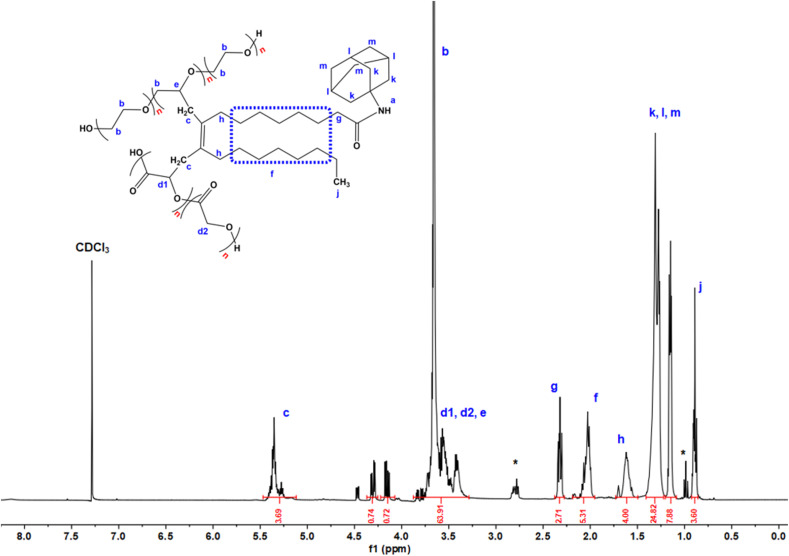
Proton NMR spectrum of amantadine/OA-Pluronic F68-PLGA micelles. The key chemical shifts are labelled to identify various proton environments in the system. The signal at ∼3.5 ppm corresponds to methylene groups in Pluronic F68, while the peak at ∼1.25 ppm is attributed to the methylene protons of oleic acid. Other notable peaks include the amantadine protons observed at ∼1.5 to 2.5 ppm, indicating its successful encapsulation. The spectrum highlights the characteristic shifts corresponding to different chemical environments in the micellar structure, providing evidence of the interaction between the hydrophobic core (oleic acid and PLGA) and the hydrophilic segments (Pluronic F68 and PLGA).

Proton NMR (^1^H NMR) analysis provided detailed information about the molecular interactions within the micelles (Fig. 4). The amantadine encapsulated in the micelles showed characteristic chemical shifts, including deshielding of the methylene (CH_2_) protons in the amantadine ring at 4.0 ppm, attributed to the electronegativity of the amide bond formed during encapsulation. The primary amine group's signal was observed at 7.2 ppm, further indicating successful incorporation of amantadine into the micellar core. The characteristic peaks of the triblock copolymer Pluronic F68 (PPO) were observed in the range of 1.0 ppm to 3.5 ppm, with the methyl group (–CH_3_) at 1.3 ppm and the methylene (–CH_2_) group at 3.8 ppm. These shifts highlight the interactions between oleic acid, Pluronic F68, and PLGA, confirming the stability and structural integrity of the micelle system.

### 3.2. Controlling amantadine release from micelles

**Fig. 5:**
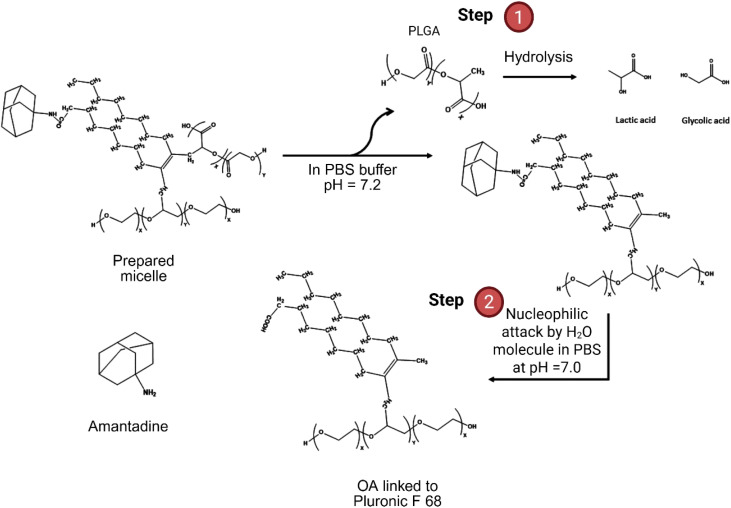
Schematic representation of the degradation mechanism of amantadine-loaded micelles in PBS buffer at pH 7.2. The PLGA backbone undergoes hydrolysis by water molecules, leading to ester bond cleavage and releasing lactic acid and glycolic acid (step 1). This is followed by a nucleophilic attack by the water molecules to the amide bond facilitating the release of amantadine from oleic acid and Pluronic F68 (step 2).

**Fig. 6:**
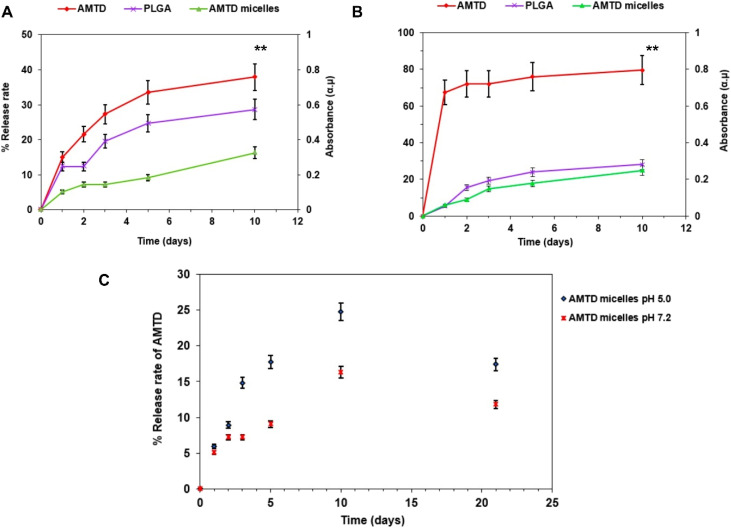
Drug release profiles of amantadine drug, PLGA, and amantadine-loaded micelles under various pH conditions. (A and B) Release rates of amantadine, PLGA, and encapsulated amantadine at different pH conditions, pH 7.2 and pH 5.0, respectively over ten days. (C) A comparative release rates of encapsulated amantadine in PBS, highlighting faster release rates at mildly acidic pH 5.0 than neutral pH 7.2. The (**p < 0.01) represents the significant variation of statistical analysis performed using one-way ANOVA.

Encapsulated amantadine release from micelles was determined using PBS buffer solution at two different pH conditions: circumneutral (pH 7.2) and slightly acidic (pH 5.0). The structure of the micelles made by oleic acid, Pluronic F68, and PLGA, showed slow release rates due to their hydrophobic properties compared to standard amantadine, as demonstrated in Fig. 5 and 6. The mechanism of amantadine drug release from micelles is graphically shown in Fig. 5. The outer shell formed by PLGA undergoes hydrolysis due to nucleophilic attack of the ester bond caused by absorbed water molecules, breaking down PLGA into lactic acid and glycolic acid (Step 1). Upon degradation of PLGA, the hydrophilic segments (PEO) in Pluronic F68 increases the porosity of micelles and facilitates the migration of amantadine from the hydrophobic core (oleic acid), by breaking the amide bond due to nucleophilic attack caused by water molecules and a reduction of the strength of van der Waals forces between bonds.

Fig. 6 shows a comparison of the release rates of amantadine, PLGA, and amantadine-loaded micelles in PBS buffer at pH = 7.2 (Fig. 6A) and pH = 5.0 (Fig. 6B). Herein, the release rate of encapsulated amantadine indicated by measuring the absorbance of samples added to buffer solution at different time intervals 0, 1, 2, 3, 5, 10, and 21 days. All samples in the buffer solution at pH = 7.2 showed release rates (<50%) of the total 0.5 mg ml^−1^ added to the PBS, within 21 days. Encapsulated amantadine showed maximum release rate at 16.3% over the same period of time (Fig. 6A). In comparison, encapsulated amantadine in PBS buffer (pH = 5.0) demonstrated a maximum release from micelles (24.8%) of the total concentration (0.5 mg ml^−1^) added to PBS buffer (Fig. 6B). A significant difference in release rate was observed for just the amantadine drug compared to PLGA and encapsulated amantadine, showing maximum release rate in acidic conditions (79.6%) compared to neutral (37.9%). This difference in release rates is linked to the polymer's chemical structures and properties, which are sensitive to pH changes. These factors impact polymer degradation, water penetration and ultimately the diffusion of the encapsulated drug. The release rate profile significantly reduced at the limit of 11.8% (pH 7.2) and 17.4% (pH 5.0) after 10 days and did not show any further release of amantadine beyond this point (Fig. 6C). Previous studies demonstrated that factors such as cross-linking and hydrophobic interactions may impact or reduce drug release rates from polymeric micelles.^13,26–28^

Our micellar system aimed to add Pluronic F68 to stabilize the micelles and to achieve greater accumulation in the brain by facilitating transport across BBB endothelial cells. Pluronic-amine block copolymers significantly improve the delivery of polypeptides across the blood–brain barrier and promote their accumulation in the brain, both *in vitro* and *in vivo*. In our current study, the focus was primarily on developing a micelle-based sustained release platform and characterizing its release behaviour *in vitro* at different pH values.

### 3.3. Cytotoxicity

The cytotoxicity of the amantadine-loaded micelles was evaluated using the MTT and SRB assays, both of which measure cell viability by different mechanisms. The MTT assay reflects mitochondrial activity *via* the reduction of tetrazolium salts to formazan crystals in viable cells, while the SRB assay quantifies cell viability by protein binding. Both assays were conducted on J774 macrophage cells at various concentrations of free amantadine and amantadine encapsulated in the micelles.

**Fig. 7:**
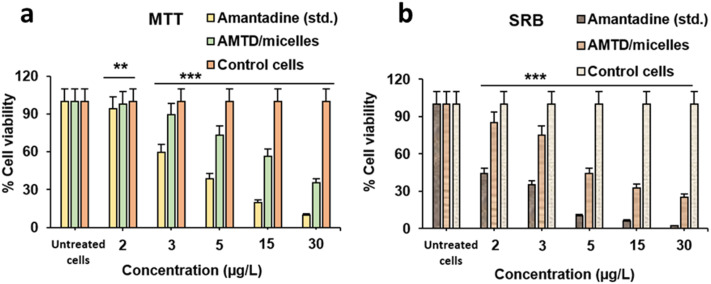
MTT (a) and SRB (b) *in vitro* cytotoxicity assays of amantadine drug and micelles on J774 cell line macrophages. The results are expressed as % of cell viability (mean ± Stdev.) *versus* control cells in triplicates (*n* = 3). Statistical analysis was performed using one-way ANOVA (**p < 0.01, ***p < 0.001).

The MTT assay (Fig. 7a) showed a clear concentration-dependent decrease in cell viability for both free amantadine and micelle-encapsulated amantadine. At higher concentrations, such as 15 μg L^−1^ and 30 μg L^−1^, the free amantadine exhibited significant toxicity, reducing cell viability to 19.8% at 15 μg L^−1^ and below 10% at 30 μg L^−1^. In contrast, the micelle-encapsulated amantadine significantly reduced the toxicity, with cell viability reaching 56.5% at 15 μg L^−1^ and about 30% at 30 μg L^−1^. At lower concentrations (2 μg L^−1^ and 3 μg L^−1^), encapsulated amantadine caused minimal cytotoxicity, reducing cell viability by only 10.7% at 3 μg L^−1^ compared to over 40% reduction with free amantadine.

The SRB assay results (Fig. 7b) corroborate the findings from the MTT assay. The free amantadine caused a severe reduction in cell viability at concentrations of 5 μg L^−1^ and above, with over 90% cell death at concentrations ranging from 5 μg L^−1^ to 30 μg L^−1^. However, micelle-encapsulated amantadine significantly improved cell viability, with 85% and 74.9% cell viability at 2 μg L^−1^ and 3 μg L^−1^, respectively. Even at higher concentrations, encapsulated amantadine consistently showed reduced toxicity compared to the free drug.

These results clearly demonstrate that encapsulating amantadine within the PLGA micelles substantially mitigates the cytotoxic effects seen with free amantadine. This reduction in toxicity is likely due to the controlled and sustained release offered by the micelle system, which prevents the high local concentrations of the drug that can induce cytotoxicity. Moreover, the protective nature of the micelle structure likely shields the drug from rapid degradation and interaction with cellular components, as previously noted by ref. 29 and 30. The improved cell viability with micelle-encapsulated amantadine also indicates better biocompatibility, making this formulation more suitable for therapeutic applications, particularly in neurodegenerative diseases such as Parkinson's.

These findings are consistent with the overall goal of the study, which aimed to improve drug delivery and reduce systemic toxicity using a dual-surfactant system composed of Pluronic F68 and oleic acid. The sustained release profile, coupled with the improved cytocompatibility of the micelles, enhances the potential of this formulation to provide safer, more effective treatment for Parkinson's disease. Further studies, particularly *in vivo*, would be beneficial to fully assess the therapeutic efficacy and long-term safety of this micellar drug delivery system.

The structural stability and controlled release capabilities of the micelles, as indicated by the DLS, TEM, FTIR, and NMR data, directly correlate with the improved cytotoxicity profiles observed in the MTT and SRB assays. Encapsulating amantadine within the micelles significantly reduced its cytotoxicity at higher concentrations (15 μg L^−1^ and 30 μg L^−1^), compared to the free drug. The protection offered by the hydrophobic core–shell structure of the micelles further enhances biocompatibility, shielding the drug from premature release and interaction with cellular components, which aligns with previously reported results.^29,30^

The combination of Pluronic F68 and oleic acid as surfactants, along with the biodegradable PLGA polymer, has demonstrated a novel and effective approach for amantadine encapsulation, resulting in stable micelles with reduced cytotoxicity and controlled release. This study not only validates the potential of this formulation for improved drug delivery in Parkinson's disease but also opens new avenues for the encapsulation of other therapeutics. The stability, biocompatibility, and reduced toxicity observed with the micelles present a promising strategy for advancing drug delivery systems, offering a safer and more effective therapeutic option for patients.

## 4. Outlook

This study demonstrates the potential of PLGA micelles, enhanced by Pluronic F68 and oleic acid, to improve amantadine delivery in treating Parkinson's disease. The formulation's stability, reduced cytotoxicity, and controlled drug release are promising, but further research is needed to fully explore its therapeutic potential. Future work should focus on conducting *in vivo* studies to assess pharmacokinetics, biodistribution, and therapeutic efficacy in animal models, determining if the micelles can effectively cross the blood–brain barrier and maintain therapeutic levels. Additionally, long-term stability and degradation studies under physiological conditions are necessary to understand how the PLGA matrix degrades and influences drug release kinetics. Optimization of micelle size could further enhance delivery efficiency, particularly in crossing the blood–brain barrier, through fine-tuning the surfactant and polymer concentrations. Another exciting avenue for future research is exploring combination therapies by co-encapsulating amantadine with neuroprotective or dopaminergic agents, potentially leading to synergistic effects for treating neurodegenerative diseases. Furthermore, the versatility of the micellar system suggests potential applications beyond Parkinson's disease, such as in treating other neurological disorders or conditions that require controlled drug release. By addressing these areas, this micellar formulation could be refined for clinical translation, offering a novel approach for enhancing drug delivery and reducing side effects in therapeutic applications.

## Author contributions

Ismail A. Adwibi: writing – original draft, methodology, investigation, formal analysis, data curation, conceptualisation. Swaroop Chakraborty: writing, review & editing, methodology, investigation. Bashiru Ibrahim: writing, review & editing, methodology, investigation. Hanene Ali-Boucetta: supervision, review & editing, methodology, conceptualisation, supervision. Eugenia Valsami-Jones: writing, review & editing, validation, conceptualisation, funding, supervision.

## Conflicts of interest

There are no conflicts to declare.

## Supplementary Material

RA-015-D5RA05261K-s001

## Data Availability

All data analysed by dynamic light scattering measurements, FTIR/NMR spectra, drug release profiles, and cytotoxicity assay are included in this published article and available upon request. Supplementary information is available. See DOI: https://doi.org/10.1039/d5ra05261k.
