# Supplementary material for: Enhancing amantadine delivery through PLGA micelles: a novel approach using oleic acid and Pluronic F68 for sustained release and reduced toxicity
Source: RSC Adv. 2025 Nov 4;15(50):42630–9. doi: 10.1039/d5ra05261k (PMC12584735; doi:10.1039/d5ra05261k)
Supplement: RA-015-D5RA05261K-s001 [file RA-015-D5RA05261K-s001.pdf]

Supplementary Information (SI) for RSC Advances.
This journal is © The Royal Society of Chemistry 2025

Supplementary information

Drug loading amount in micelles:

The amount of drug-loaded in micelles calculated as following:

Data analyzed using UV-Vis spectroscopy. The calibration curve of standard amantadine was prepared by measuring the absorbance at various concentrations of amantadine (figure S.1)

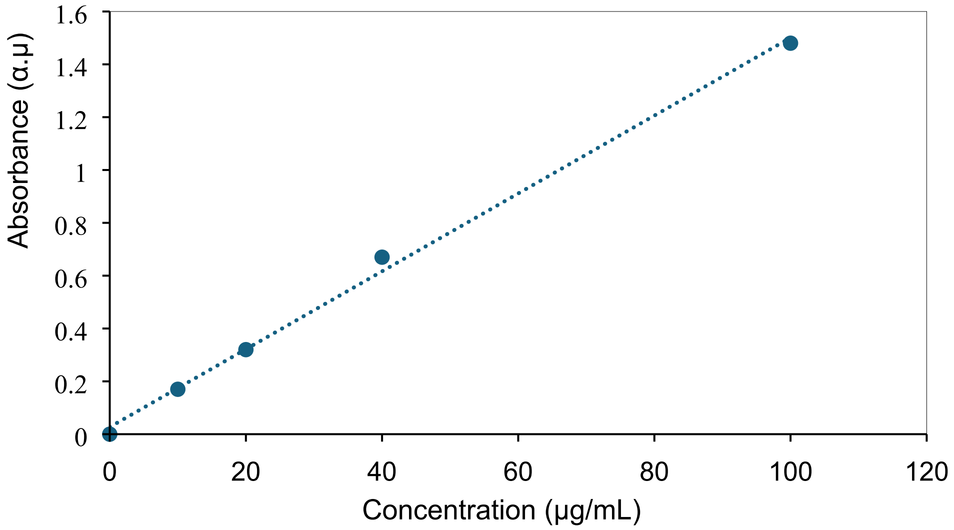

Figure S.1 Calibration curve of standard amantadine obtained using UV–Vis spectroscopy. Absorbance was recorded at the characteristic wavelength of amantadine across a series of known concentrations 0, 10, 20, 40, and 100 μg/mL.

The absorbance of unencapsulated/free amantadine drug in the supernatant (for 3 washes) was indicated at 0.045, 0.021, and 0.003

Total free amantadine in the supernatant: 0.045+0.021+0.003 = 0.069

(Supernatant analysis determines how much drug was *not* encapsulated)

However,

To calculate the unencapsulated amantadine from the calibration curve:

$y = 0.0147x + 0.0269$

$x = 0.069 - 0.0269 / 0.0147 = 2.86$ μg/mL * 15 mL (5mL each wash) = 42.9 μg = 0.043 mg

Therefore,

Drug loaded amount = (Total amount of drug added – unencapsulated drug in the supernatant) = 2.5mg – 0.043 mg = 2.457 mg, which equal 98.28% w/w

Or

Drug loading efficiency:

$$\% \, EE = \frac{weight\ of\ drug\ in\ micelles}{Weight\ of\ drug\ initially\ added} \, x\, 100$$

$$\% \, EE = \frac{2.457\ mg}{2.5\ mg} \, x\, 100$$

1H NMR analysis

The Pluronic F68, Amantadine, and PLGA were also analyzed as demonstrated in figures S2, S3, and S4, respectively.

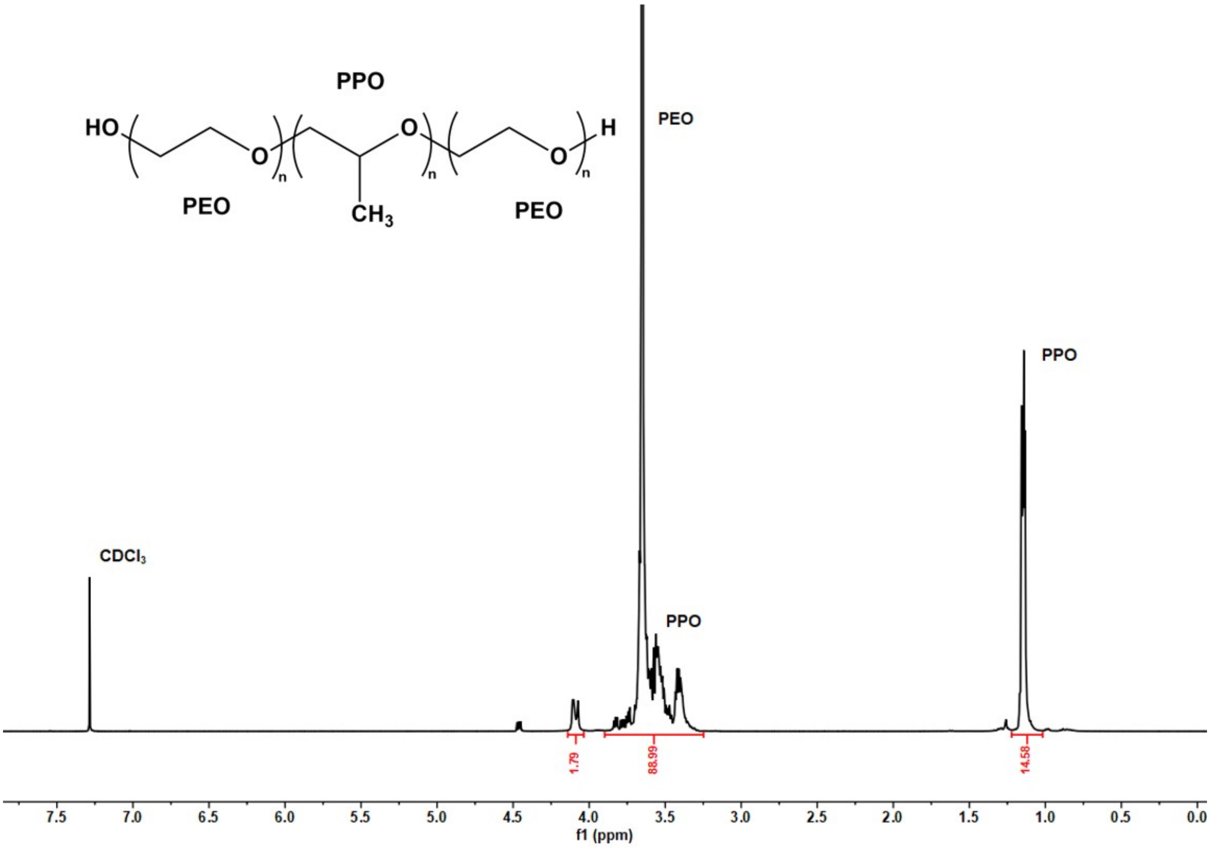

Figure S2: ¹H NMR spectrum of Pluronic F68 dissolved in $CDCl_3$. The solvent peak appears at around 7.26 ppm. Characteristic resonances of the hydrophobic polypropylene oxide (PPO) blocks are observed at around 3.5 ppm (–CH–CH_2–) and 1.1 ppm (–CH_3), while the strong signal at 3.6 ppm represents the hydrophilic polyethylene oxide (PEO) blocks.

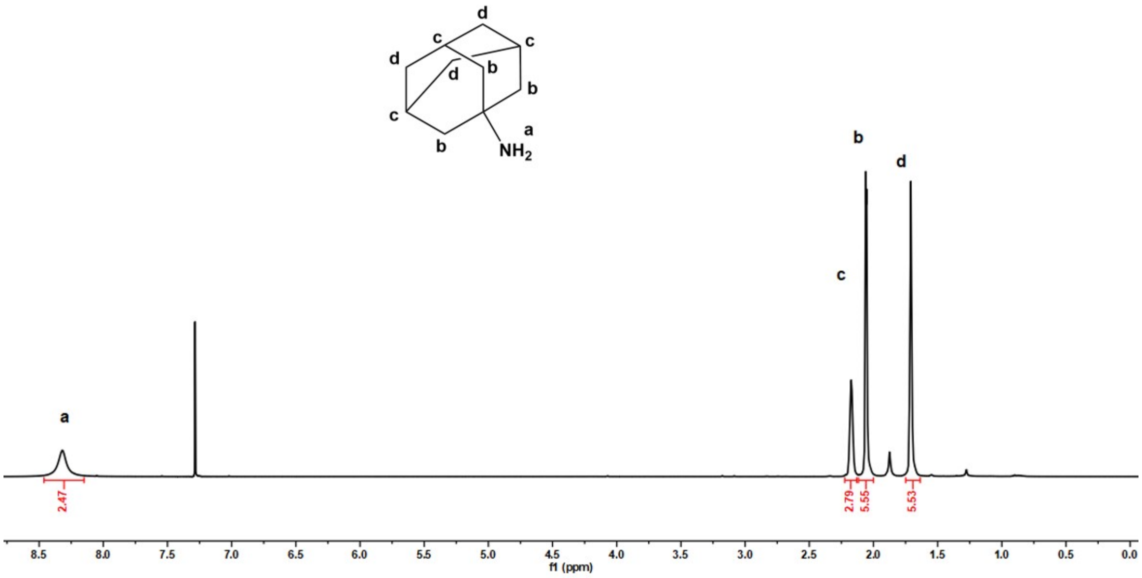

Figure S3 ^{1}H NMR spectrum of amantadine. The proton of amine group (a) resonated at 8.5 ppm, while the cage hydrogens appear as distinct signals in the region between 1.6 and 2.2 ppm for methylene (b, c) and methine (d) protons.

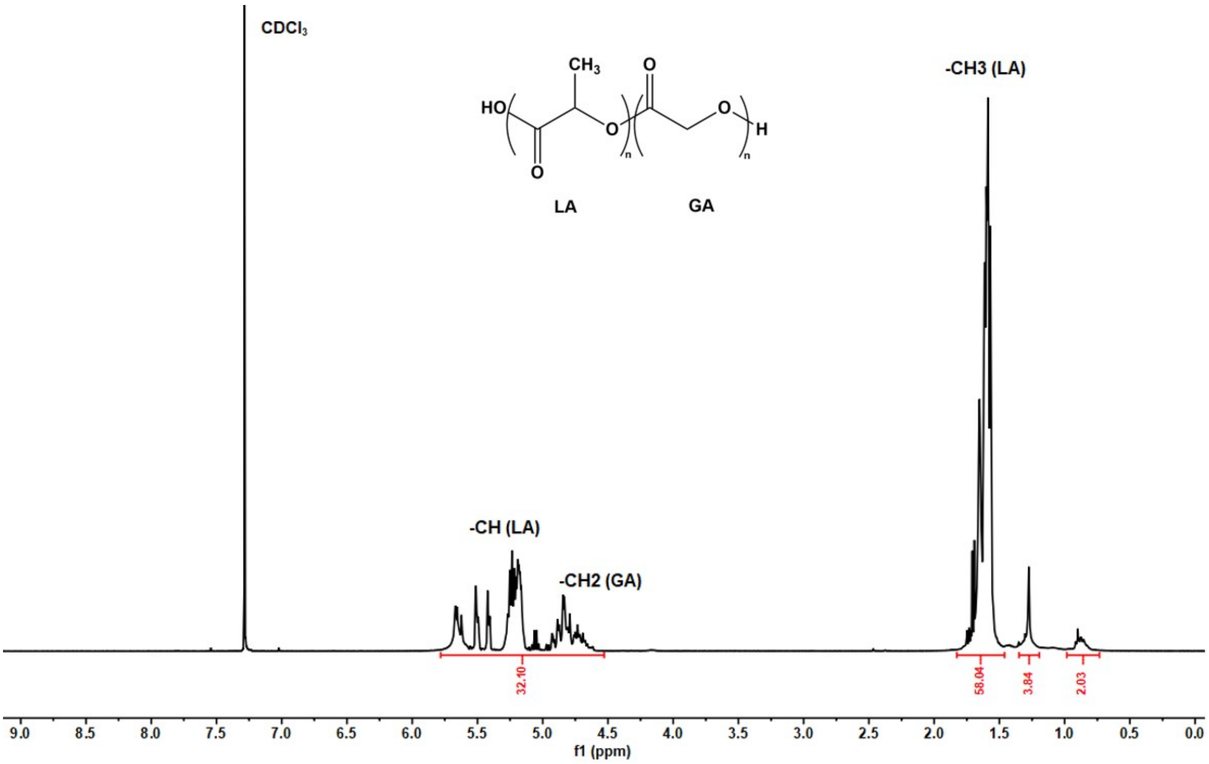

Figure S4: ^{1}H NMR spectrum of poly (lactic-co-glycolic acid) (PLGA). The methine proton of lactic acid units (LA) appears at 5.2 ppm, while the methylene protons of glycolic acid units (GA) resonated at approximately 4.7 ppm. The methyl protons of lactic acid give a strong signal at 1.6 ppm. The characteristic peaks confirm the presence of both lactic acid and glycolic acid segments in the copolymer structure.

Distortionless Enhancement by Polarization Transfer (DEPT-135) (^{13}C NMR) analysis of Pluronic F68, PLGA, and Pluronic F68–Amantadine/OA–PLGA micelles

Different types of carbon atoms in Pluronic F68, PLGA and Pluronic F68–Amantadine/OA–PLGA micelles was analyzed using 13CNMR (DEPT 135) as shown in Figure S5:

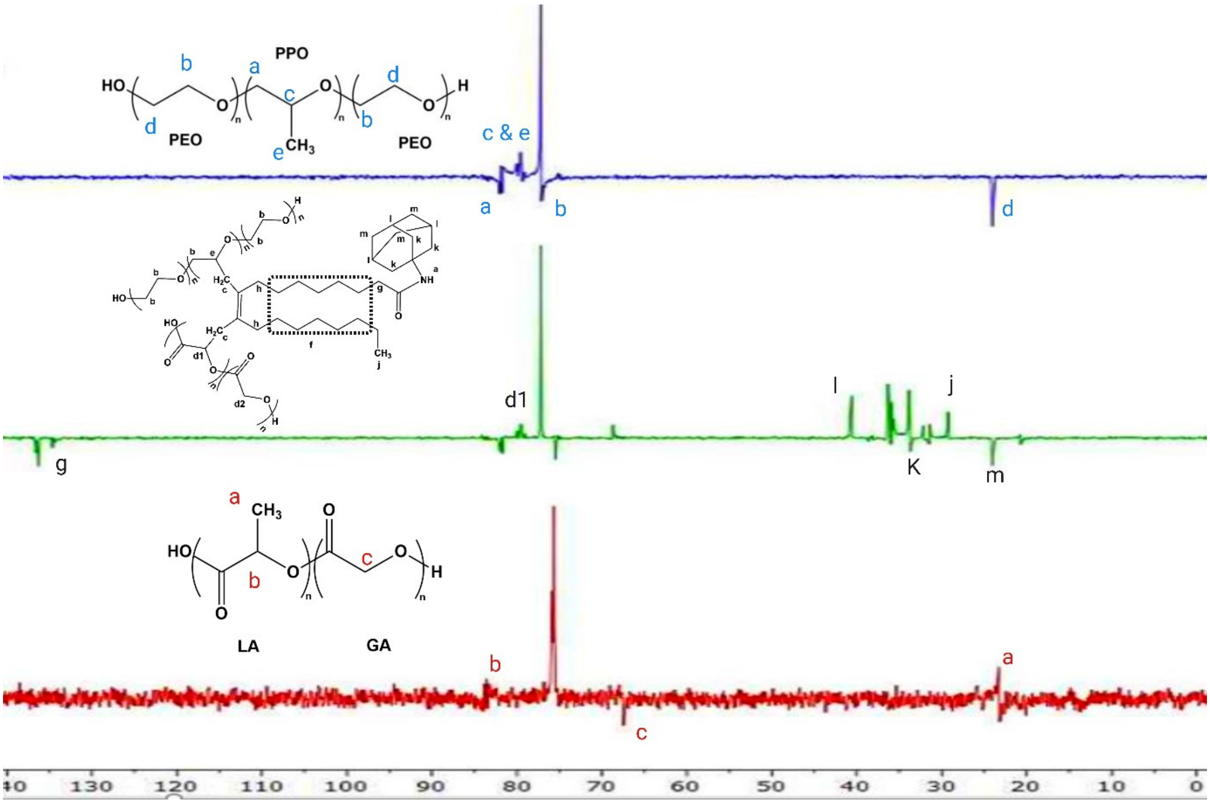

Figure S5: 13 C NMR (DEPT 135) spectra of Pluronic F68 (top, blue), AMTD micelles (middle, green), and poly (lactic-co-glycolic acid) (PLGA) (bottom, red). CH and CH_3 groups shown as positive signals, whereas (CH2) as negative signals.
